# Napoleon a Sainte Helene. Opinion d'un Medecin sur la Maladie de l'Empereur, et sur la Cause de sa Mort, &c.

**Published:** 1829-04-01

**Authors:** 


**NAPOLEON A SAINTE HELENE. OPINION D'UN MEDECIN SUR LA MALADIE DE L'EMPEREUR NAPOLEON, ET SUR LA CAUSE DE SA MORT ; OFFERTE A SON FILS, AU JOUR DE SA MAJORITE. Par. *S. Hereau*, Ancien Chirurgien de Madame Mere, et Premier Chirurgien de l'Emperatrice Marie Louise. 8vo. pp. 228. Paris. 1829.**

"Je meurs prematurement, assassine par l'Oligarchie Anglaise et son sicaire." *Testament de Napoleon.*

And left a name, at which the world grew pale,
To point a moral or adorn a tale. *Johnson.*

NAPOLEON at St. HELENA, in 1829 ! ! Be not alarmed, gentle reader, with the idea that we are turning from sober science to the "ROMANCE of HISTORY." Although there is no other branch of science or literature so intimately connected with morality—or, in one word, with a knowledge of human nature, as is the practice of medicine; yet we have rarely digressed into what are considered the proper provinces of the DIVINE and the PHILOSOPHER. Neither shall we trespass far on the confines of these reverend and sage personages, on the present occasion, though there is ample scope—and even some excuse for such digression. The work at the head of this article was in our possession some months before we determined to notice it—and we should not have introduced it to the attention of our readers, had we not observed that almost the whole of the French periodical press (medical) has yielded implicit credence and assent to the facts and conclusions contained in M. Hereau's book. In no one instance have our Continental or Transatlantic cotemporaries accused us of national prejudice or narrow-minded partiality—and when we bring to bear on the work before us, the stern dictates of reason, and the calm deductions of medical philosophy, we shall probably shake to their centre some of those gorgeous fabrics erected by political bias, heated imagination—and (what should never enter into medical discussions) national antipathy !

We do not deem it incumbent on us to agitate the question respecting the wisdom or policy of our Government in consigning NAPOLEON to St. Helena. After what took place at Elba, it was evident that the EX-EMPEROR could not be trusted at large—and we should be glad to know how he would have liked to be locked up in the Tower of London, to be gazed at with the other lions of that place ? Had Napoleon ended his days in the Tower, we should have had whole volumes descriptive of the *insalubrity* of that dreadful prison. The exhalations from Billingsgate, the dripping damps of father Thames, the abominable smell of tar from the ships, the everlasting din of drunken sailors, joined with the foggy atmosphere of London, would have afforded more materials for the TRISTIA of the Bonapartists, than Ovid ever found on the banks of the Euxine ! No spot on the face of this globe would or could have pleased Napoleon, *as a prison ;* and no man who dreaded universal war and all its dire consequences, could have thought of leaving that warrior at his liberty. Of St. Helena itself we shall have occasion to speak presently.

When the news of Napoleon's death reached Paris, in July 1821, it was currently reported, and almost universally believed, that the Ex-Emperor had been put to death by violence. Some said a scuffle had ensued between Sir Hudson Lowe and Napoleon, and that the latter was assassinated—others, that the illustrious prisoner had been enticed to the edge of a precipice and then hurled over—that he had been shot—smothered, &c. &c. But the report which gained most credit, and lasts till this hour, was, that Napoleon had been slowly poisoned, by order of the British Government, and through the instrumentality of Sir H. Lowe ! !* It is to clear up these doubts and to investigate the real cause of Napoleon's death, that the volume before us has been written. After a most laborious inquiry, the author arrives at the conclusion, that the late EMPEROR was neither poisoned, shot, nor hurled over a precipice; and as we believe that no one, on this side of the Channel, will doubt the correctness of these conclusions, we shall not enter into the negative arguments and data on which they are founded.† His affirmative conclusions appear to be, that the death, "de ce grand homme," was effected by three series of causes—the insalubrity of St. Helena—the ignorance of the English doctors—and the cruelty of Sir Hudson Lowe—the latter cause including, of course, a host of moral affections, as chagrin, grief, resentment, hope deferred, blasted ambition, &c. &c. &c.

Among the causes of death which were bruited about at the period in question, one was, that the Emperor had poisoned himself by means of a deadly drug, which, like Hannibal, of old, he always carried about with him. M. Hereau brings forward a document little, if at all, known in this country, shewing (if it be authentic) that Napoleon nearly effected suicide at Fontainbleau, just before his abdication. The poison appears clearly to have been prussic acid.

In the second chapter of the work, our author inquires into the grounds of the opinion, once prevalent, that Napoleon died of a disease hereditary in his family—CANCER OF THE STOMACH. He decides in the negative. It was reported, but not, he thinks, proved, that the Emperor's father died of the above disease. Be this as it may, none of the brothers or sisters of Napoleon had any complaint of the kind; and M. Hereau denies that the disease in Napoleon's stomach was of this description. He informs us that Bonaparte lived on the most simple aliments, and always fell in with the diet of the country where he happened to sojourn—contrary to the habits of his most distinguished gene-

---

* The author of the volume acknowledges that he himself was a believer in the story of *poisoning*, till he investigated the whole history of Napoleon's exile.

† At page 10 and 11 the author plainly avers that the conduct of the British Minister, for the Foreign Department, the manner in which the Ex-Emperor was entrapped ("*la maniere dont on s'etait saisi de sa personne*") the plan of exile, the spot of imprisonment—and, above all, the character of the jailor, fully authorised all and every suspicion of murder which could possibly be spread on the occasion ! ! The whole of the French medical journals re-echo these base insinuations against our Government and country! Is it not wonderful that, with all the superlative penetration and talent of Napoleon, so lauded by his partisans, he should have been so infatuated and blind as to trust himself in the hands of the English ? This piece of oversight is not once alluded to by our illustrious confrere, M. Hereau ! For our own parts, we entertain a very different opinion of Napoleon's sagacity. His life would not have been worth three weeks' purchase, had he not made his escape in the Northumberland to the shores of his oldest and most decided enemy.

rals, who, in the wilds of Russia, took care to have the wines of Spain, and the ices of Italy on their tables. It is certain that, while in Egypt, he contracted a cutaneous affection (affection dartreuse) which never entirely disappeared till the battle of Wagram. On that memorable field of his glory, he was exposed for days and nights to cold and wet. The eruption disappeared, feverishness, pains in the head, and various other symptoms of a repelled eruption supervened. For these, the celebrated Frank and Corvisart were consulted. The former thought seriously, the latter very lightly of the complaint. The fact is certain that, from the time of this repulsion, the Emperor was affected with attacks which were represented by some as epileptic, and by others as of an apoplectic character. At St. Helena the cutaneous complaint returned, especially upon the thighs; but the principal infirmity under which Bonaparte laboured, and which he often predicted to be the ultimate cause of his death, was a dysuria, which commenced before the Russian campaign, and was productive of excessive sufferings. He was often heard to say—" *C'est la ma partie foible! C'est par la que je perirai*"! In this prognostication he was deceived—and, considering the slow and dreadful death resulting from diseases of the bladder and urinary passages, Napoleon was probably fortunate in being carried off by a disease which occasioned very little suffering.

We now come to that chapter of the work where the question is agitated whether or not the climate of St. Helena was sufficient to produce the disease under which the Emperor succumbed? It is some 22 years since we examined, with a critical eye, the medical topography and natural scenery of St. Helena, during a short stay at that island, on our return from the East Indies—and our sentiments respecting it are on record.* We hesitate not to assert that, if the whole surface of this globe were examined, there could hardly be found a spot of equal extent, presenting so many features of the sublime and beautiful as the calumniated island of St. Helena.

Rising abruptly to the height of nearly 3000 feet above the surface of an ocean that is never ruffled by a storm, and where the South-east breezes have blown with undeviating course since the deluge, the island in question presents the picture of desolation to the wondering mariner, while approaching this summit of some huge ante-diluvian Alp—"girt with a chain of inaccessible precipices."† The ship steers within pistol-shot of rocks a thousand feet in height—not merely perpendicular, but actually overhanging the "murmuring surge" that chafes, and foams, and frets against the adamantine barrier below. No indication of human, or even vegetable existence is perceived in any direction; when suddenly the scene changes, and every crag is seen bristling with men and cannon. A narrow fissure in the stupendous wall of rock, formed by some convulsion of the earth, discloses a crowded town between two gigantic

---

* When Dr. Johnson's description of St. Helena, as published in the "Oriental Voyager," was shewn to Napoleon, the Emperor demurred to the fidelity of the portrait, observing that *he* could not see the beauties of the scenery, as there delineated. It requires but little insight into human nature to know how very differently the same objects are viewed by a traveller, full of youthful emotions, and an exiled prisoner, hurled from the throne of his ambition to finish his days on an insulated rock in the midst of a vast ocean! It is but fair to say that Napoleon never had an opportunity of seeing the beautiful parts of the island on which his bones repose.

† Oriental Voyager.

precipices, opening a zig-zag path to the interior of the island. Nothing but the arid rock and volcanic lava can be seen around this coast of desolation; but this gloomy and forbidding barrier passed, the most romantic and fairy scenes that poet's imagination ever fancied, burst, in succession, on the astonished and enraptured sight. The view of Sandy-Bay valley from Sandy-Bay ridge or from Diana's Peak, surpasses, in romantic and picturesque beauty, though not perhaps in sublimity, any thing which the Alps, Appennines, or Pyrennees present. Had Samuel Johnson's eye been familiar with this valley, he might have drawn, from Nature, a more enchanting abode for Rasselas, than his pen has done from the utmost flight of his fancy!*

In respect to the salubrity or insalubrity of St. Helena, the inquiries which we made, ten years before Napoleon's arrival there, joined with an examination of its medical topography, convinced us that there were few places on the earth more compatible with longevity, under ordinary circumstances. It cannot be doubted, however, that the degree of salubrity differs in different parts of the island. A sudden elevation of 3000 feet in the middle of a great ocean must naturally cause much concentration of vapour around the higher peaks, with corresponding fogs and even rain, while the vallies are dry and clear. The situation of Longwood, therefore, was both unfortunate and injudicious —unfortunate for the credit and the humanity of the British nation and the governor—injudicious, inasmuch as there could be no possible necessity for selecting one of the most disagreeable spots in an impregnable fortress like Saint Helena, for the confinement of any prisoner. It appears that Napoleon enjoyed good health while at the Briars, a few miles from James Town; but soon after being transferred to Longwood, he got dissatisfied in mind and disordered in body. The wretched and crooked policy which dictated Napoleon's removal from the Briars, confinement at Longwood, and perpetual annoyance there, has, of course, cast a stain on our country which time can never efface—and we envy not the feelings of that man, whose conduct sickened the heart and revolted the feelings of every inhabitant of the civilized world.

Not only Napoleon, but those who accompanied him, the soldiers and the sailors, became sickly, and great numbers of the navy and army died. The circumstance of the sailors becoming unhealthy, would induce a suspicion that some of those inscrutable causes of insalubrity, which occasionally render the most delightful spots of the earth the scenes of desolating disease, happened to come into operation at the time of Napoleon's exile at St. Helena, and thus procured for that romantic isle a character for unhealthiness which it did not generally deserve. Be this as it may, we do not deny that *physical* causes

* Vide Oriental Voyager. Even M. Hereau, who draws a frightful picture of St. Helena, is forced to confess that—"la vue dont on jouit sur la chaîne de SANDY BAY, et sur le sommet de DIANA's PEAK, est sublime. Tantôt l'on aperçoit de verts paturages—des jardins ornés d'arbres—des maisons construites dans la vallée, ou sur le penchant des hauteurs, ce qui offre a l'oeil fatigué de la vue des precipices affreux et des ravins de couleur rougeâtre du voisinage, un agréable soulagement. Ces contrastes font trouver au spectateur la partie cultivée de l'isle pittoresque et romantique."

The above is nearly a literal translation of a passage in Dr. Johnson's description of St. Helena in the year 1806, and may reconcile the discrepancy of opinion between a traveller's and a prisoner's conception of things.

operated on the health of Napoleon at Longwood, and proved auxiliaries (of greater or less power) to the host of moral causes, which were in full operation on both mind and body.

The conviction in the mind of Napoleon, and fully partaken by the author of the work before us, that the British ministry calculated, almost to a month, on the time which would be required to destroy Napoleon by the deleterious climate of Longwood, is perfectly preposterous. We are far from absolving the colonial minister from negligence of his duty, and from want of feeling; but to suppose that any English minister or ministry could calmly meditate on such murder as they are here charged with, is monstrous in the extreme.

The 4th chapter of the work is dedicated to an investigation of the disgusting restrictions imposed on Napoleon by Sir Hudson Lowe. We have not patience even to read them—and they can never be forgotton by the present generation, to whom they are but too familiar. The conduct of Sir Hudson Lowe evidently augmented the effects of the moral and physical causes previously acting on Napoleon, and his health now became visibly impaired. The sedentary life which he led—the humidity of the situation—and the perpetual vexation of mind, began to affect the digestive organs, and impair their functions. In July 1816, he complained of pain in his right side—and in September of the same year, he began to exhibit symptoms of sea-scurvy—namely, swelling and sponginess of the gums—hæmorrhages from the same—general debility and excessive lassitude, on making the least exertion. On the 29th November his legs were observed to be swelled, and the glands of the groin to be enlarged. On the 3d of December he had a rigor, succeeded by fever. On the 17th he was threatened with apoplexy—was advised to take aperients—but refused to do so, observing that he had never taken any medicine from his infancy, and he was sure that a purgative would be destructive to him. On the 24th March, 1817, Mr. O'Meara's journal closes for a period, at which time we find the Ex-Emperor with swelled legs, diarrhœa, head-ache, and insomnolency. A hiatus of six months ensues, during which, we are informed that the time was passed in alternations of head-ache, pains in the limbs, defluxions on the gums, insomnium, dyspepsy. On the 25th September (1817) we find Napoleon affected with nausea. On the 1st of October, Mr. O'Meara states that he complained of dull pain in the right hypochondrium, as also at the top of the shoulder of that side—slight disposition to cough—restlessness at night—spongy gums—œdematous legs. Mr. O'Meara pronounced a somewhat doubtful or hesitating diagnosis of HEPATITIS, at this time;—and afterwards, our author affirms, this was the prevalent idea among the prisoner's medical attendants. On the 3d October, the right side was found to be enlarged—the appetite still farther diminished—the legs œdematous—inclination to vomit, from time to time—great anxiety of countenance, and watchfulness. On the 26th January, 1818, constipation and symptoms of dyspepsia—inclination to vomit—flatulence—continual pain in the right side.

During the succeeding six months, our author informs us that the symptoms continued to increase, "under the administration of remedies of a more and more irritating kind"—"medication de plus en plus perturbatrice."* On the

* M. Hereau appears to be a worthy disciple of the school of *radico-liberals* in this country, who are free from every tie of honour and of medical ethics—and who

25th July, 1818, Mr. O'Meara quitted St. Helena. M. Hereau here recapitulates the symptoms of hepatitis, which have been alluded to, and argues that they were more indicative of a *general inflammatory state of the abdominal viscera*, and ought to have led to general and local depletion, instead of "le traitement excitateur," which the fatal error of routinism put in force.

Now we think there can be no doubt that the symptoms which we have described indicated local depletion, at least from the epigastric region; but we cannot help censuring the subterfuge which M. Hereau makes use of, in calling gentle purgation an "excitant treatment." It is just as much a depletive measure as leeching; and we have no doubt that, had the late Napoleon been an obedient and tractable patient under Mr. O'Meara, Mr. Stokoe, and the other medical attendants, it would have been much better for him, and he would now, probably, have been in the land of the living.

Mr. O'Meara, on his return to Europe, is reported to have made strong representations to the relatives of Bonaparte, and, in consequence thereof, two physicians were selected by Cardinal Fesch to repair to St. Helena. It has often been remarked that, whenever any want of success attended the French arms, some sinister accident was called in to account for the failure. So in this case. M. Hereau laments that two of the most eminent physicians of Paris (who would, he says, have been delighted with the expatriation in so noble a cause) were not selected, instead of "deux hommes obscurs," of whom, one was a priest, who had merely studied the principles of medicine for a few years! We suspect that very few of the eminent Parisian physicians would have volunteered upon this occasion. Be this as it may, M. Hereau enters here into a most severe critique on the medical report which Mr. O'Meara sent to ROME, and which was there taken into consideration, before the medical commissioners departed for St. Helena. It is not our intention to examine this critique. It is probable that Mr. O'Meara did not design it as a strictly *medical* case; but rather as a *political* one, in order that attention might be excited to the deplorable situation in which he left Napoleon. In a council of physicians who took Mr. O'Meara's statement into consideration at Rome, a code of instructions was drawn up, containing a series of puerile, or rather anile precepts, dietetic, disciplinal, and therapeutic, which we shall not impose on the patience of our readers. We may remark, however, that it was equally harmless and useless, whatever might be the nature of the complaint.

Messrs. Vignali and Antommarchi having arrived in London, had the good fortune to meet Mr. O'Meara, who described to them, *viva voce*, the situation of their future patient. Mr. Stokoe, who had only attended Napoleon for a few days, after the departure of Mr. O'Meara, represented the royal patient as in a state of extreme debility, labouring under violent pain in the region of the liver, darting thence to the right shoulder—severe pain in the head about midnight, with vertigo, &c. On the second day's visit Mr. Stokoe prescribed venesection and a brisk purgative. The latter was refused—but the former was complied with; and the patient was much relieved by it. Mr. S. now examined carefully the region of the liver, and expressed his conviction that

---

conceive that the great object of a medical man is to traduce his neighbour, both publicly and privately, without any proof or evidence—since, in this consists the true LIBERTY OF THE PRESS and of the subject!!

this organ was seriously affected—"gravement affecté." He therefore recommended mercurials.

M. Hereau passes a severe sentence on the reports of Mr. Stokoe, during the few days which he attended on the Ex-Emperor, and also on the naval surgeons of Great Britain, whom he supposes as—"restés forcément et bien malheureusement, par la nature de leurs fonctions, peu au courant des progrés de l'anatomie pathologique qui, depuis vingt ans, a fait faire de si grands pas a la medécine en France."

We shall not imitate the illiberality of M. Hereau, on this occasion. We have always given credit to his countrymen for the efforts they have made towards the advancement of medical science; but we believe that, in this country, where the state of the case must be best known, no one would be hardy enough to accuse the naval medical officers of being behind their brethren, whether in the army or of private life, as far as regards medical science. It is well known that the medical officers of our fleets and armies have contributed very materially, during the last thirty years, to the advancement of pathological as well as therapeutical science. The sweeping censure of M. Hereau is as unjust as it is illiberal.

Messrs. Vignali and Antommarchi did not arrive at St. Helena till the 18th September, 1819. They appear to have taken great pains to consult all the LIVER DOCTORS in London, and a curious consultation is given by M. Hereau, for the guidance of the Italian physicians; but as there is no actual signature, we shall not criticise it. It is highly ridiculous and melancholy to observe the tax which is paid by the great men of the world, when their lives are in danger. They have not half the chance of rational treatment which the veriest pauper enjoys in a dispensary or hospital! So many formalities to be gone through—so many conflicting opinions of relatives to be reconciled—so many prejudices, as to this and that physician, to be overcome—and, after all, when a whole conclave of MEDICOS are gathered together, it is often the most superficial and opiniated individual who gives the tone to the consultation, and all chance of steady systematic measures is annihilated by the glorious prerogatives of rank and wealth! Be it so! It is probably a wise dispensation of Providence that riches and titles—especially the latter, should pay a tribute that brings them pretty much on a par with their more humble neighbours.

On the 23d September Antommarchi was admitted to the presence of the fallen Emperor. He was lying on a camp-bed—his face pallid and of an earthy hue—eyes sunken—conjunctiva of a reddish yellow colour—body extremely fat—tongue slightly furred—dry cough with viscid expectoration—abdomen rather hard—pulse small, but regular, and 60 in the minute. On a more particular examination, M. Antommarchi discovered that the *left lobe of the liver was indurated and extremely tender on pressure—gall-bladder turgid with bile, and salient near the cartilage of the third rib.* The patient complained of vague pains and uneasiness in the right side and loins, as well as in the right shoulder. Pressure at the scrobiculus cordis occasioned difficulty of breathing. Napoleon complained of pain, varying in intensity, in the right hypochondrium, which he described as seated about two inches from the surface—the appetite was gone—nausea and even vomiting obtained. The urine was natural—there were copious daily perspirations.

This report of Antommarchi undergoes a severe, but not a very fair criticism by the author of the work. There are some portions of the description, especially that passage which we have marked in Italics, that are open to animadversion; but, upon the whole, we do not much wonder that the disease was considered as one of the LIVER, considering how much the mind of the observer had been pre-occupied, we might say prejudiced, by this idea. It is much more easy for a man, after dissection, to criticise the diagnosis formed by others during life, than to form a correct one himself, if placed under similar circumstances. It appears that Napoleon was not very particular as to his diet, and that he generally drank half a bottle of claret daily. This regimen is, of course, blamed tremendously by M. Hereau, who appears to be an ultra-disciple of Broussais, and who look supon the disease, from beginning to end, as neither more nor less than the veritable GASTRO-ENTERITE of the Broussaian school.

Antommarchi directed a stimulant and anodyne liniment to the epigastric and hypochondriac regions. He also suggested mercury, both internally and externally—" but, fortunately, the Emperor refused to comply." Heat and fever became augmented. At this time, Bonaparte was in the habit of walking to a spring in the neighbourhood of Longwood, where he delighted to quench his thirst. At this spring he desired to be buried, and his request was complied with. The following passage will shew the feelings of the Bonapartists, even to this hour.

> "The body has, in fact, been deposited there, and is guarded with as much care as when it was a living body—not to prevent profanation of the tomb; for every kind of insult was offered to the Emperor while in captivity:—No! it is the fate of the assassins of Cæsar which Napoleon's enemies dread! They well remember that, at the sight of the bleeding corpse of Cæsar, a whole people rose in arms to avenge his death!"

However fair the comparison between Cæsar and Napoleon may be, as far as the millions which they destroyed by their ambition may be concerned, we do protest against the analogy between the assassination of Cæsar and the death of Napoleon. What medical man, unbiassed by political feeling, could take upon him to say, that the death of Napoleon was positively caused by the climate of St. Helena, or the irritating conduct of Sir Hudson Lowe? Do we see no people die, in the course of four or five years, unless they have been exiled to an island in the Atlantic, or under the suveillance of a British General:—Yet the sentiments of M. Hereau are received with acclamation by the whole of the French medical press! We raise our voice against these conclusions, not entirely for the sake of our national honour, but in the cause of truth and true philosophy;—and our voice will, fortunately, be heard in every region which "the rising or the setting sun surveys."

But to return. On the 17th December, 1819, M. Antommarchi states, that "*the pain in the liver was insupportable*"—and that there were symptoms of enteritis. A hiatus of *seven months* then occurs in the medical journal, during which, no mention is made of the state of Napoleon's health! On the 18th July, 1820, the Doctor writes to his friend, the Chevalier Colonna, at Rome, as follows:—

> "I have now been ten months in attendance on Napoleon. I found him affected with chronic hepatitis, of the most severe kind. My remedial measures appeared to have been crowned with success. The Emperor was fast recovering—took exercise—and was projecting the formation of a garden around his habitation—but my hopes are blasted, for the influence of climate, the *proximate cause* (cause prochaine) of chronic hepatitis is too powerful for the constitution of our illustrious patient, and baffles all remedies."

Dr. A. goes on to state, that the Emperor had had a relapse, attended with ardent fever, acute pain in the liver, and also in the right ankle, with erysipelas of that limb, which symptoms he attributed to disorder of the digestive organs and of the hepatic functions. In all these phenomena, M. Hereau, on the contrary, sees nothing but the clearest symptoms of gastritis, with its attendant sympathetic affections, such as the inflammation of the ankle-joint.

On the 17th March, 1821, Antommarchi writes again to the Chevalier Colonna, that, since his last report, the hepatic disease had gained ground, and produced general disorder of the digestive organs. The patient could now only take small quantities of liquid nourishment, and even these were sometimes rejected by vomiting. On the 21st March, a small dose of emetic medicine was given, and next day the abdomen became swelled, accompanied with a paroxysm of ague fever, great oppression at the epigastrium, and sense of suffocation, from the great quantity of glairy fluid secreted from the alimentary and aerial passages. The patient demanded and took an emetic. The following day there was a severe paroxysm of fever, with icy coldness of the lower extremities—meteorism of the abdomen (tympanitis)—obstinate constipation. The emetic was again repeated, and followed by the same accident. On the 25th March, Dr. Arnott, of the 20th Regiment, advised a large blister over the abdomen—to exhibit strong purgatives, &c. This advice was rejected by the Emperor, because it was English practice. Lavements, however brought away large quantities of dense and glutinous matters. The Emperor's aversion to any thing in the shape of medicine, however simple or palatable, was truly astonishing! Heaven help the faculty in this country, should Napoleon's taste ever become the prevailing one. They might shut up shop at once! "C'est une chose inouïe que l'aversion que je porte aux medicaments. Je courais les dangers avec indifference—je voyais la mort sans emotion—et je ne peux quelque effort que je fasse, approcher de mes levres un vase qui renferme la plus légère preparation!" Bonaparte was not only a spoiled child of FORTUNE, but also of PHYSIC! He had hardly ever taken a dose of Doctor's stuff, from infancy till the time of his last illness.

We find some trifling medicines, however, exhibited, and gastric irritability increase. On the 10th April, Dr. Arnott, after an examination of the royal patient, affirmed that the disease was not in the liver, but in the stomach. Saline effervescing medicines were now ordered, with opiates. The vomiting continued, with much pain in the gastric and intestinal regions. On the 18th April, he refused to take any more physic, and made a speech to Dr. Arnott, which is full of poignant accusations towards our government, and especially—"l'infame Hudson Lowe." On the 28th of the same month, the Emperor began to agree with the opinion of Dr. Arnott, contrary to that of Antommarchi, that the stomach was the seat of organic disease; and he desired that his body might be examined after death. The vomitings were very frequent, and the Emperor expressed an opinion that he was dying of the same disease which killed his father—scirrhus of the pylorus. "Doctor," said he, "take particular care to examine this part—put your remarks on paper, and send them to my son. I could wish to guard him, at least, from this disease." On the 2d of May, we find the royal sufferer delirious—and on the 5th his mortal career ceased!

We will not follow M. Hereau through the train of bitter reflexions which he pours on the medical attendants, and especially on M. Antommarchi, whom he loads with curses for his ignorance and obstinacy. We shall proceed at once to the *post-mortem* examination, avoiding, however, a great deal of useless detail respecting unimportant parts, and also the phrenological admeasurements of the Emperor's head, put on record by Antommarchi.

*Dissection.* Besides the Generals Bertrand and Montholon, there were present Drs. Short, Arnott, Mitchell, Livingstone, and several other medical gentlemen.

The body was considerably emaciated—not being one-fourth the size it was when Antommarchi arrived on the Island. The abdomen was very much distended. The left side of the chest contained a few ounces of clear yellow serum, and the costal and pulmonary pleura was covered with coagulable lymph. The superior lobe of this lung was studded with tubercles, and there were some small tubercular excavations. There was rather a larger quantity of clear effusion in the right side of the chest. The right lung was sound. The mucous membrane of the trachea and bronchia was red and inflamed. There was nothing particular about the pericardium, heart, or great vessels.

There was much gas in the cavity of the peritoneum, and a soft transparent exudation covered the surface of this membrane, parietal and intestinal. The liver and spleen, indurated (durcis), were very large and gorged with blood, (tres volumineux, &c.)—but the structure of the liver did not present any notable organic change. The gall-bladder was distended with thick and viscid bile. "The liver, which was affected with chronic hepatitis, was intimately adherent, on its convex side, to the diaphragm, by means of strong and old membranes." The concave surface was strongly adherent to the stomach. The stomach itself appeared externally quite sound, exhibiting no trace of phlogosis. At the distance of three inches from the pylorus, however, where the stomach was strongly adherent to the liver, the coats of this organ were completely penetrated by an aperture. On opening the stomach, some glairy, acrid, and coffee-coloured matters were found in its cavity—and these being washed out, the mucous membrane was observed to be natural, between the small and the great cul-de-sac of this organ—"depuis le petit jusq'au grand cul-de-sac de ce viscere." Almost the whole of the rest of the internal surface of the stomach was occupied by one vast cancerous ulcer, "ulcere cancereux," destructive of all, except the peritoneal coat of the organ. The pyloric extremity of the stomach was scirrhous, to the extent of an inch from the pylorus. The opening, at this part, was natural. The little epiploon was indurated and very much degenerated. The lymphatic glands of this part were enlarged, scirrhous, and some of them suppurated. The mucous membrane of the intestines was healthy. There was no other disease, cognizable by the senses, in the body.

Such is the post-mortem examination, as recorded by Antommarchi. Another and much shorter account is given by the English medical attendants, differing, in some essential points, from that of the Italian physician.*

Viewing the author of the work before us as a special pleader, or an advocate, whose *object* (leaving *duty* out of the question) was to make all circumstances

* They tell us that, with the exception of adhesions to the stomach and diaphragm, the liver presented no morbid appearance, and they say nothing whatever respecting enlargement of that organ, or of the spleen. On the other hand, they tell us, that "almost the whole internal surface of the stomach presented one mass of cancerous affections, or of scirrhous parts changing into cancer, especially in the neighbourhood of the pylorus. The cardiac extremity *only*, and that of very small extent, was sound."

tend to support one particular point of view, we determined, like an impartial judge, to weigh the facts or evidence, without listening at all to the arguments of the pleader, and to come to a conclusion, uninfluenced by declamation or prejudice. We, therefore, made up our mind, *before* we read the commentaries of M. Hereau, lest we should be drawn aside by specious or sophistical ratiocination.

It is evident, from the accounts published by both parties, that the Emperor died of the disease in the stomach. Nor is there any proof, even in Antommarchi's version of the dissection, of HEPATITIS. The term "*tres volumineux*," is a mere comparative expression, and is contradicted by the English examiners who assert that "le foi ne presentait rien de malsain." In respect to the function of the liver, we have no doubt that it was disordered ; but it is not a little strange that so many medical men in succession should fall into such delusion, day after day, and month after month, regarding the imaginary disease of the liver ! ! The nature of the disease of the stomach can scarcely admit of a doubt. Both parties agree in describing the disease as of the cancerous and scirrhous character. Into a long discussion with M. Hereau respecting GASTRITIS as the veritable nature of the complaint, we shall not enter. Neither shall we attempt to persuade him (for it is quite impossible) that this said gastritis, allowing it to be such, was not caused by the incendiary and murderous treatment of the English medical attendants. M. Hereau has no doubt about the matter—although he records the almost total abstinence from physic which Napoleon observed till a late period of his disease! As for scirrhus or cancer of the stomach, in this case, our author treats the idea with sovereign contempt. "Who is the physician," says he, "that could fail to recognise in the appearances above-mentioned, the *ordinary effects of chronic inflammation* kept up by perpetual excitation." Without entering into the litigated doctrines of hereditary diseases, we confess that we can see nothing in the *post mortem* examination, or in the living phenomena here recorded, which induces us to refer the disease of the illustrious patient exclusively to gastritis, independently of any disposition to that specific and morbid process denominated scirrhus or cancer. We are quite willing to allow that the host of moral and physical causes, of a depressing character, which had been operating on the mind and body of Napoleon, long before he was expatriated to Saint Helena, must have had a powerful influence in deranging the functions, not only of the stomach, but of all the other auxiliary organs of digestion. Still, if we consider the great temperance of the Ex-Emperor—his abstinence not only from all spirituous liquors, but even from medicine ; we cannot bring our minds to the Broussaian doctrine, that inflammation—simply inflammation of the mucous membrane of the stomach and bowels was the necessary result of these moral and physical causes. If, on the other hand, we bear in mind the many instances which present themselves in practice, or are recorded in the fasti of medical science, where grief, and the other depressing passions call into activity the disposition to, or actually produce scirrhus and cancer of the stomach, we shall be inclined to conclude that the talents and fortune of Napoleon did not exempt him from the laws which govern and bind human nature in the humble as well as in the ambitious walks of life !

The case is extremely interesting in a medical as well as moral point of view. The remarkable tenacity with which both the English and the Italian medical attendants clung to the idea of HEPATITIS, and by which prepossession they overlooked the more fatal malady going on in the STOMACH, is deserving of record, though we cannot join in the acrimonious censure which M. Hereau has poured on them. As we said before, there were several symptoms which might readily lead astray the practitioner, and induce him to think they were attributable to disease of the liver, when the extensive sympathies of the stomach were the real agents and actors in the drama. It must also be conceded, that the gastric phenomena were comparatively faint—or, at all events, they were much less prominent than might have been expected, till a late period of the disease. The inflammation in the chest—that in the membranes of the liver, and even in the peritoneum, was not accompanied by manifest or corresponding symptoms. Perhaps the character of Napoleon—his scepticism as to the powers, and his aversion to the taste of medicine, contributed to veil the whole case in a cloud of mystery which would not have attended a similar complaint in the ordinary race of mortals.

In philosophical reflexions on this occasion we dare not indulge; else we should be inclined to point out the eventful life and melancholy death of NAPOLEON as affording the most instructive moral lesson that has ever been recorded on the page of history! The magic eye of imagination cannot help glancing back on the career of this wonderful man, from his humble birth on a miserable island in the Mediterranean, up to the possession of Europe's sceptre—and down again to the captive's wretched cell and sepulchre amid the barren crags of a rock, a thousand miles from any habitable shore! When we picture to ourselves his dawning military genius at Toulon—his daring and decided politics in the storms of the Revolution—his Cæsarian ambition in assuming the purple—his rivalry of Hannibal in urging an army, with heavy artillery, over the frozen and apparently impassable summits of the very Alps crossed by the Carthaginian General—

> "So when proud ROME the Afric warrior braved,
> "And high on Alps his crimson banners waved;
> "Though rocks on rocks their beetling brows oppose,
> "With piney forests and unfathomed snows—
> "Where girt with clouds the rifted mountain yawns,
> "And chills with length of shade the dewy lawns,
> "Onward he marched to LATIUM's velvet ground!"

his Alexandrine bravery on many a bloody field, as well as his Alexandrine temerity, in warring against the elements themselves—

> "On Egypt's burning sands and Russia's snows"—

when (we say) we picture to our imagination these, and many other scenes of Napoleon's "strange eventful history," we are apt to be dazzled with the splendour of his arms and the lustre of his fame! But PROVIDENCE has not permitted such a fascinating picture to be held up to man's ambitious view without a reverse. The halo of glory around Napoleon, which excited amazement and envy throughout the world at large, was consuming the soul of the hero with anxiety—perhaps with fear;—and the man who conquered myriads of veterans on the field of Austerlitz, was urged to commit midnight murder on the disarmed and defenceless DUC D'ENGHIEN!

> Non enim Gaza, neque consularis
> Submovet lictor miseros tumultus
> Mentis, et curas laqueata circum
> Tecta volantes!

But what signifies an individual life! The poisoned sick at Jaffa—the frozen army on the plains of Moscow, are but as mere specks in the galaxy of havoc and destruction which marked the path of one ambitious man! Is it then wonderful—is it inconsistent with retributive justice, or an over-ruling providence, that the cause of so many wars, of such waste and expenditure of human life,—that Napoleon the destroyer, (for every great warrior is a destructive angel) should taste the bitter cup of suffering before he quitted the stage? Is he who fertilised the soil of Europe, for 20 years, with the blood of its inhabitants, to be held up as an object of universal commiseration, because he was insulated from general society, in a place where he could do no harm—because the atmosphere of St. Helena was not as bright as that of Italy—because his house of exile at Longwood was not as commodious as Versailles—because it was not, or rather (without meaning a pun) because it *was* a MAL-MAISON! We believe, indeed, that Napoleon performed in this world, an exemplary expiation for whatever crimes he may have committed;—but we are convinced, at the same time, that his sufferings, mental and corporeal, were principally owing to himself. If he had been half as much the PHILOSOPHER in adversity, as he was the HERO in prosperity, he might have ended his days, and very much protracted them, even at St. Helena, by scorning the petty insults of his narrow-minded jailor, and by dedicating the remainder of his life to literary recreations and a record of his eventful career. Instead of exhibiting this true PHILOSOPHY—this true HEROISM, we find the conqueror of Europe fretted about every the most trifling concern of his little establishment—quarrelling about his wine, his beef, his rations, with as much intensity as a discontented sailor in a ship of war:—pouring forth his lugubrious grievances about provender, &c. &c. to Mr. O'Meara and M. Antommarchi, with as much—nay, with far more solemnity than he formerly pronounced the dethronement of kings, and the extinction of nations! Was this in conformity with the precepts of philosophy of which he boasted so much?

> Lætus in presens animus, quod ultra est
> Oderit curare, et amara lento
> Temperet risu. Nihil est ab omni
> Parte beatum.

The disease and death of Napoleon are worthy of record on the page of medical history—while his life and captivity will furnish ample materials for the pens of the politician, historian, philosopher, and divine. We have performed our part of the task.

